# Both overlapping and independent mechanisms determine how diet and insulin-ligand knockouts extend lifespan of *Drosophila melanogaster*

**DOI:** 10.1038/s41514-017-0004-0

**Published:** 2017-02-20

**Authors:** Jelle Zandveld, Joost van den Heuvel, Bastiaan J. Zwaan, Matthew D.W. Piper

**Affiliations:** 1 Laboratory of Genetics, Wageningen University and Research Center, 6708 PB Wageningen, The Netherlands; 2 Institute for Cell and Molecular Biosciences, Newcastle University, NE4 5PL Newcastle Upon Tyne, UK; 3 Department of Genetics, Institute of Healthy Ageing, Evolution and Environment, University College London, London, UK; 4 Present Address: School of Biological Sciences, Monash University, Clayton, Australia

## Abstract

Lifespan in many organisms, including *Drosophila melanogaster*, can be increased by reduced insulin-IGF-like signaling (IIS) or by changes in diet. Most studies testing whether IIS is involved in diet-mediated lifespan extension employ only a few diets, but recent data shows that a broad range of nutritional environments is required. Here, we present lifespan data of long-lived *Drosophila*, lacking three of the eight insulin-like peptides [*Drosophila* insulin-like peptides 2,3,5 (*dilp2-3,5*)] on nine different diets that surround the optimum for lifespan. Their nutritional content was varied by manipulating sugar and yeast concentrations independently, and thus incorporated changes in both diet restriction and nutrient balance. The mutants were substantially longer-lived than controls on every diet, but the effects on the lifespan response to sugar and yeast differed. Our data illustrates how a greater coverage of diet balance (DB) and restriction can unify differing interpretations of how IIS might be involved in the response of lifespan to diet.

## Introduction

Lifespan in many organisms, including the fruit fly *Drosophila melanogaster,* can be increased by genetically reducing activity of the IIS pathway and also by a reduction of food intake without malnutrition (dietary restriction, DR).^1–3^

Typically, DR involves the use of two or more diets that represent increasing severity of restriction. The lifespan response to these treatments traces an inverted U-shape with relatively short life at high food concentrations, ascending to a peak at intermediate food levels, and decreasing again as nutrients become limiting and the organism increasingly suffers from starvation. When analyzing how a long-lived mutant modulates this response, several interacting effects are possible,^4^ but two parameters are thought to be key, (1) does the mutation alter maximum life expectancy attained across all nutritional conditions, and, (2) is there a change in the shape of the response of lifespan to diet? A mutation that attains the first can be argued to extend life, at least in part, through a mechanism independent of DR since it builds on a state in which diet induced longevity is presumed already maximized. However, if a mutation shows the second type of change then it can be interpreted to mediate at least part of the DR response and thus that DR and the mutation are mechanistically linked.

Reports vary on whether or not IIS and DR extend life through an overlapping or independent mechanism.^2, 3, 5–7^ Reduced IIS by knockout of *Drosophila* insulin-like peptides (*dilps),* the fly’s homologs for human insulin, has been proposed to mediate the benefits of DR because dietary yeast concentration affects the relative expression of *dilp5*.^5^ Moreover, knocking out three (*dilp2-3,5*) of the eight *dilp*s extends lifespan and reduces the magnitude of lifespan change in response to DR^3^—similar to what is observed when overexpressing a dominant negative form of insulin receptor (*InR*).^6^ However, other studies indicate that IIS and DR affect lifespan independently; flies without the IIS transcription factor *dFOXO* or lacking the insulin receptor substrate (*chico*) still demonstrate significant lifespan changes in response to DR and do not necessarily extend lifespan beyond that of controls on DR.^2, 5, 7^ Thus it is not yet clear to what extent IIS signaling and DR interact and through what mechanism.

Often, the specific set of ingredients and the practices adopted to impose DR are not identical between laboratories^8^ and so DR in one laboratory is likely to be nutritionally different from that in another. Recent studies have revealed that not only the quantity but also the ratio between dietary protein and carbohydrates can account for the lifespan effects observed under DR.^9, 10^ Thus, IIS-by-DR interaction studies could yield different outcomes because the mutation may alter the response of lifespan to some nutritional components, but not to others. If true, apparently contradictory outcomes of how a mutant affects the DR response can be resolved when they are viewed as distinct parts of a single lifespan response surface in multidimensional nutrient space.^4^

To explore this potential explanation, we measured the lifespan of long-lived *dilp2-3,5* mutants and control flies on nine food types, representing all combinations of three yeast and three sugar concentrations (50, 100 and 200 g l^−1^ each). These diets cover the range of foods used for past studies of IIS-by-DR interactions but extend them by incorporating changes in both DB and diet restriction (DR).

## Results and Discussion

**Fig. 1:**
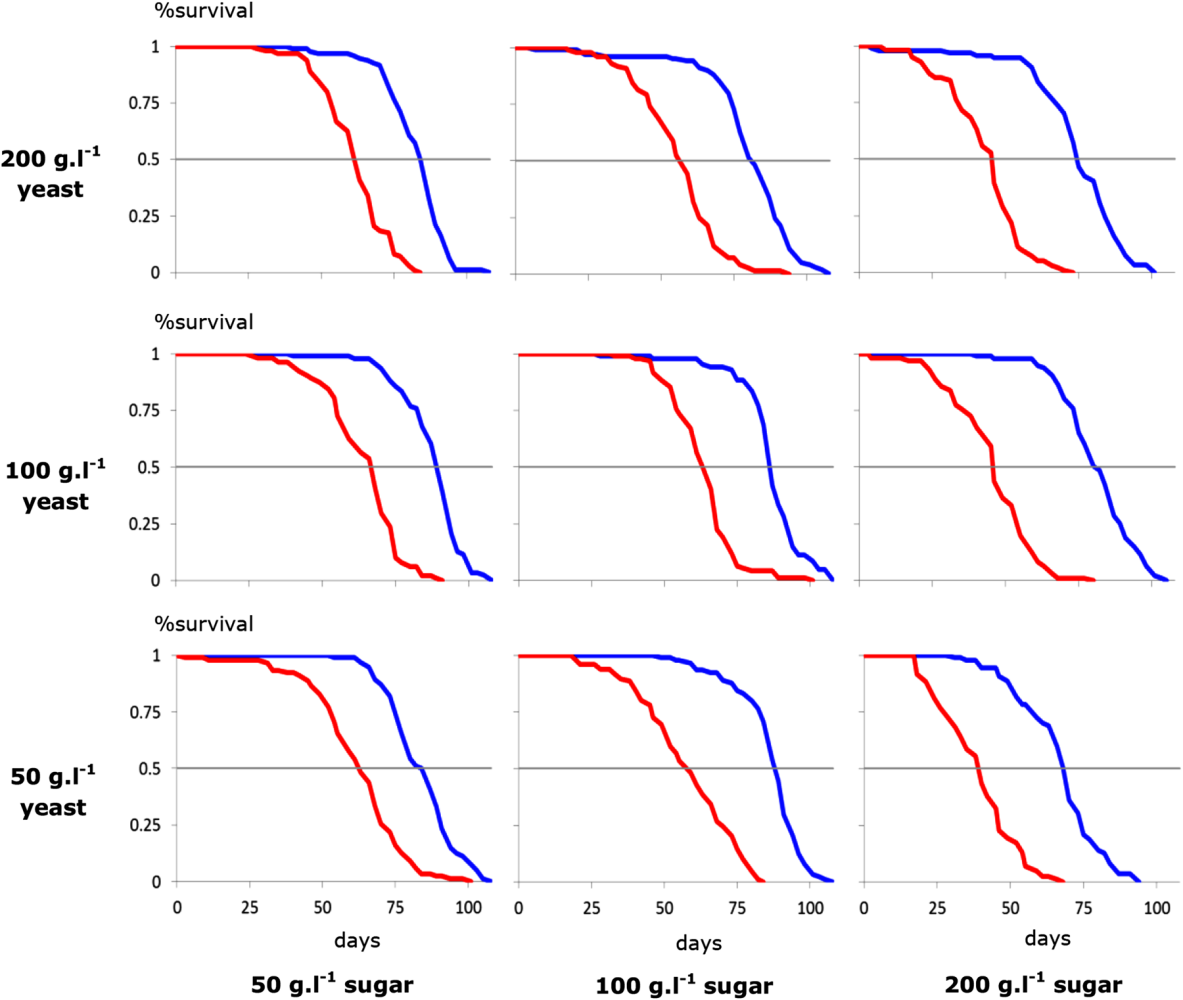
Lifespan curves for *dilp2-3,5* mutant (*blue*) and control flies (*red*) for all nine food types. On each row the lifespan response to one level of dietary yeast is shown for three different levels of sugar (50, 100 and 200 g l^−1^, respectively)

**Fig. 2:**
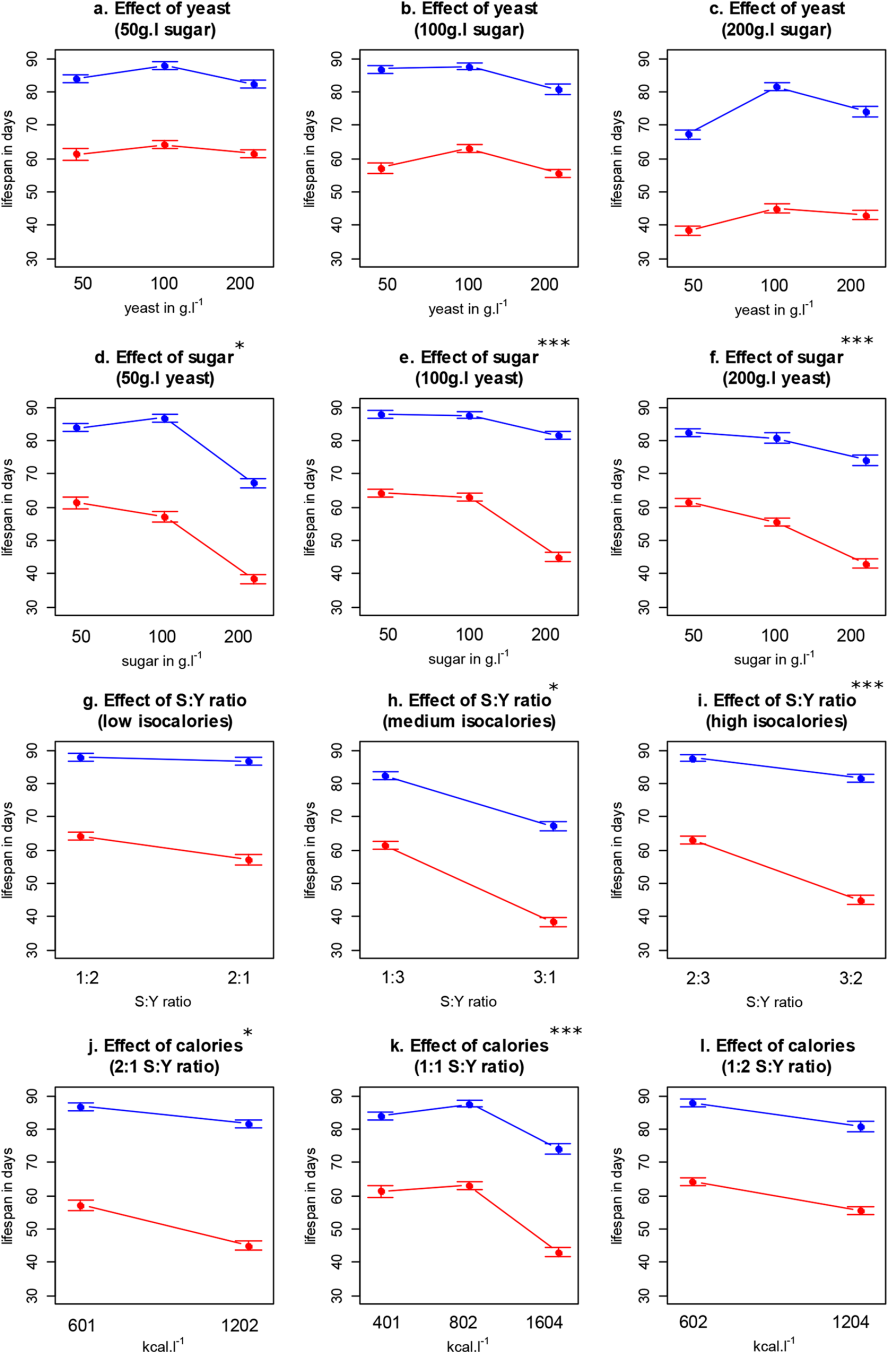
Lifespan interaction plots (mean +/− standard error) display how the lifespan response to nutritionally different DR interventions can be differently affected by the knockout (*blue lines* represent *dilp2-3,5* mutants, *red lines* control flies), **a**–**c** effect of dietary yeast on different sugar levels; **d**–**f** effect of dietary sugar on different yeast levels, **g**–**i** effect of S:Y ratio on different caloric levels; **j**–**l**, effect of calories on different S:Y ratio’s. *Asterisks* indicate a significant interaction between the *dilp2-3,5* knockout and diet regimen under consideration (coxph). *p < 0.05, **p < 0.01, ***p < 0.001

We found that female *dilp2-3,5* mutants were longer-lived than controls by at least 21 days on each of our nine food types (Figs. 1, 2, Fig. S1, Table S1), including the S:Y combination at which lifespan peaks for our control flies.^9^ We also found for each diet that reproduction of control flies was higher than that of the mutants (Fig. S2, Table S2). Because *dilp2-3,5* deletion extended lifespan beyond the value of maximal wild-type longevity attainable through diet manipulation, we conclude this triple knockout extends life, at least in part, by a mechanism independent of that invoked by diet.

The optimal DB was not different for mutants and controls, and both genotypes responded similarly to all nutrient manipulations (three-way sugar-yeast-genotype interaction, *p* > 0.1, cox-proportional-hazard, coxph, Table S2). Genotype did not affect the response of lifespan to yeast (*p* > 0.1, coxph, Fig. 2a–c), but there was a significant effect of sugar such that high concentrations caused a less severe reduction in lifespan in mutants compared to controls (coxph, *p* < 0.001, Table S2, Fig. 2d–f). Thus, *dilps 2, 3* and *5* are required for the lifespan reducing effect of increasing sugar levels. Our data thus shows that both the modification of diet and deletion of *dilps 2, 3* and *5* can modify lifespan, and that the mechanisms employed are in some part overlapping, and in some part independent.

This understanding is, however, not clear when we restricted our analysis to important subsets of our diets that represent typical DR experiments. For example, when we compared the effects on lifespan of different subsets of diets the mutants could either reduce the response to DR (Fig. 2e, f, j), enhance it (Fig. 2d, k) or leave it unchanged (Figs. 2a–c, l). This same range of interaction responses was found for three sets of isocaloric diets that varied in their S:Y ratios (Fig. 2g-i). Importantly, all of these different interventions have previously been grouped under the heading “DR”, and yet they are nutritionally different and, yielding non-identical and in some cases, apparently contradictory outcomes.

Both interactive and non-interactive effects of different IIS mutations on DR have been reported^2, 3, 5–7^ and we show how these differences can be accounted for by variations in diet regimes. Another likely reason is that each IIS mutation may interact differently with DB and DR. The IIS pathway forms part of a broader nutrient signaling network, which affects lifespan in numerous ways (e.g. TOR suppression).^11^ Because each component of the canonical IIS pathway is embedded at a different point in this network they may modify the network’s overall response to diet in different ways. Further work to understand the mechanisms by which diet affects lifespan should incorporate these complex interactions between signaling networks and altered DR and DB.

## Electronic supplementary material

Supplementary Figure S2

Supplementary Figure S2.1

Supplementary Figure S2.2

Supplementary Table S1

Supplementary Table S2

Supplementary Data

## Electronic supplementary material

Supplementary Information accompanies the paper on the *npj Aging and Mechanisms of Disease* website (doi:10.1038/s41514-017-0004-0).
