# Supplementary material for: Both overlapping and independent mechanisms determine how diet and insulin-ligand knockouts extend lifespan of Drosophila melanogaster
Source: NPJ Aging Mech Dis. 2017 Feb 20;3:4. doi: 10.1038/s41514-017-0004-0 (PMC5445580; doi:10.1038/s41514-017-0004-0)
Supplement: Supplementary file 4 — Supplementary Table S1 [file 41514_2017_4_MOESM4_ESM.docx]

**Table S1. Data table phenotypes.** Per food type**, m**ean, median, standard error (se) and number of observations (n) for lifespan and reproduction for wild type and *dilp2-3,5* mutant flies.

|  |  | Yeast  (gr.l) | Sugar (gr.l) | | | | | | | | | | | |
| --- | --- | --- | --- | --- | --- | --- | --- | --- | --- | --- | --- | --- | --- | --- |
|  |  |  | 50 | | | | 100 | | | | 200 | | | |
|  |  |  | mean | median | se | n | mean | median | se | n | mean | median | se | n |
| Lifespan | wild type | 50 | 61.3 | 63 | 1.7 | 89 | 58.8 | 58 | 1.5 | 95 | 38.5 | 40 | 1.3 | 92 |
|  |  | 100 | 63.5 | 68 | 1.0 | 100 | 63.1 | 63 | 1.1 | 95 | 45.0 | 47 | 1.4 | 98 |
|  |  | 200 | 60.2 | 61 | 1.0 | 98 | 55.5 | 58 | 1.3 | 96 | 43.1 | 47 | 1.3 | 97 |
|  | *dilp2-3,5*∆ | 50 | 83.8 | 83 | 1.2 | 92 | 83.9 | 89 | 1.8 | 81 | 67.2 | 69 | 1.5 | 86 |
|  |  | 100 | 85.9 | 89 | 1.4 | 89 | 87.6 | 87 | 1.1 | 81 | 81.6 | 82 | 1.2 | 95 |
|  |  | 200 | 81.7 | 84 | 1.2 | 91 | 80.7 | 82 | 1.6 | 98 | 74.1 | 75 | 1.7 | 97 |
|  |  |  |  |  |  |  |  |  |  |  |  |  |  |  |
| Reproduction | wild type | 50 | 37.2 | 37.2 | 1.9 | 10 | 24.4 | 24.4 | 1.1 | 10 | 5.7 | 5.8 | 0.7 | 10 |
|  |  | 100 | 73.1 | 75.8 | 3.9 | 10 | 57.7 | 58.6 | 2.5 | 10 | 14.7 | 15.2 | 0.7 | 10 |
|  |  | 200 | 121.9 | 119.2 | 4.3 | 10 | 97.0 | 97.6 | 2.8 | 10 | 48.7 | 48.7 | 4.8 | 10 |
|  | *dilp2-3,5*∆ | 50 | 7.2 | 7.2 | 0.7 | 10 | 4.8 | 4.3 | 0.5 | 10 | 2.0 | 2.0 | 0.2 | 10 |
|  |  | 100 | 16.5 | 15.9 | 1.1 | 10 | 11.2 | 11.2 | 0.6 | 10 | 6.8 | 6.3 | 0.5 | 10 |
|  |  | 200 | 23.2 | 25.5 | 1.5 | 10 | 19.9 | 20.3 | 1.2 | 10 | 12.1 | 12.6 | 1.0 | 10 |
