# Supplementary material for: Both overlapping and independent mechanisms determine how diet and insulin-ligand knockouts extend lifespan of Drosophila melanogaster
Source: NPJ Aging Mech Dis. 2017 Feb 20;3:4. doi: 10.1038/s41514-017-0004-0 (PMC5445580; doi:10.1038/s41514-017-0004-0)
Supplement: Supplementary file 5 — Supplementary Table S2 [file 41514_2017_4_MOESM5_ESM.docx]

**Table S2: Statistical analyses**

**Table S2.1.** Cox proportional hazard model parameter estimates for effects of the *dilp2-3,5* knockout, increasing concentration of dietary sugar (S) and yeast (Y) and their interactions. Per factor the z-value, hazard ratio, 95% confidence interval and p-values is provided.

| *Factor* | *z-value* | *hazard ratio* | *Lower .95* | *Upper .95* | *Pr > \|z\|* |  |
| --- | --- | --- | --- | --- | --- | --- |
|  |  |  |  |  |  |  |
| *dilp2-3,5*∆ | -14.561 | 0.188 | 0.150 | 0.235 | < 2e-16 | ******* |
|  |  |  |  |  |  |  |
| S100 gr.l^-1^ | 2.108 | 1.295 | 1.018 | 1.647 | 0.035 | * |
| S200 gr.l^-1^ | 17.459 | 9.446 | 7.341 | 12.154 | < 2e-16 | *** |
| Y100 gr.l^-1^ | 0.244 | 1.030 | 0.814 | 1.303 | 0.808 |  |
| Y200 gr.l^-1^ | 2.967 | 1.430 | 1.129 | 1.811 | 0.003 | ** |
|  |  |  |  |  |  |  |
| S100 gr.l^-1^ * Y100 gr.l^-1^ | -0.507 | 0.927 | 0.692 | 1.243 | 0.612 |  |
| S200 gr.l^-1^ * Y100 gr.l^-1^ | -5.155 | 0.465 | 0.348 | 0.622 | 2.54e-07 | *** |
| S100 gr.l^-1^ * Y200 gr.l^-1^ | -0.300 | 0.957 | 0.717 | 1.277 | 0.765 |  |
| S200 gr.l^-1^ * Y200 gr.l^-1^ | -5.259 | 0.458 | 0.342 | 0.613 | 1.44e-07 | *** |
|  |  |  |  |  |  |  |
| S100 gr.l^-1^ *  *dilp2-3,5*∆ | -2.405 | 0.748 | 0.590 | 0.948 | 0.016 | * |
| S200 gr.l^-1^ *  *dilp2-3,5*∆ | -7.920 | 0.372 | 0.291 | 0.475 | 2.33e-15 | *** |
| Y100 gr.l^-1^ *  *dilp2-3,5*∆ | -1.836 | 0.799 | 0.629 | 1.015 | 0.066 | · |
| Y200 gr.l^-1^ *  *dilp2-3,5*∆ | -1.023 | 0.884 | 0.698 | 1.119 | 0.306 |  |
|  |  |  |  |  |  |  |

**S**ignificance levels: ‘·’ p<0.1 ; ‘*’ p<0.05; ‘**’p<0.01; ‘***’p<0.001

**Table S2.2.** Cox proportional hazard p-value estimates between all nine diets within genotype. Diets consisted of all possible concentrations and combinations between 50 gr.l^-1^, 100 gr.l^-1^ and 200gr.l^-1^  dietary sucrose (S) or yeast (Y).

|  |  |  |  |  |  |  |  |  |  |  |
| --- | --- | --- | --- | --- | --- | --- | --- | --- | --- | --- |
|  |  | *50S/50Y* | *50S/100Y* | *50S/200Y* | *100S/50Y* | *100S/100Y* | *100S/200Y* | *200S/50Y* | *200S/100Y* |  |
|  |  |  |  |  |  |  |  |  |  |  |
| **wild type** |  |  |  |  |  |  |  |  |  |  |
|  |  |  |  |  |  |  |  |  |  |  |
| *50S / 100Y* |  | 0.784 |  |  |  |  |  |  |  |  |
| *50S / 200Y* |  | 0.141 | 0.035 * |  |  |  |  |  |  |  |
| *100S / 50 Y* |  | 0.117 | 0.056 · | 0.822 |  |  |  |  |  |  |
| *100S / 100Y* |  | 0.461 | 0.141 | 0.525 | 0.441 |  |  |  |  |  |
| *100S / 200Y* |  | 0.003 ** | 0.000 *** | 0.037 * | 0.139 | 0.011 * |  |  |  |  |
| *200S / 50Y* |  | 0.000 *** | 0.000 *** | 0.000 *** | 0.000 *** | 0.000 *** | 0.000 *** |  |  |  |
| *200S / 100Y* |  | 0.000 *** | 0.000 *** | 0.000 *** | 0.000 *** | 0.000 *** | 0.000 *** | 0.001 ** |  |  |
| *200S / 200Y* |  | 0.000 *** | 0.000 *** | 0.000 *** | 0.000 *** | 0.000 *** | 0.000 *** | 0.046 * | 0.191 |  |
|  |  |  |  |  |  |  |  |  |  |  |

Significance levels: ‘·’ p<0.1 ; ‘*’ p<0.05; ‘**’p<0.01; ‘***’p<0.001

|  |  |  |  |  |  |  |  |  |  |  |
| --- | --- | --- | --- | --- | --- | --- | --- | --- | --- | --- |
|  |  | *50S/50Y* | *50S/100Y* | *50S/200Y* | *100S/50Y* | *100S/100Y* | *100S/200Y* | *200S/50Y* | *200S/100Y* |  |
|  |  |  |  |  |  |  |  |  |  |  |
| ***dilp2-3,5*∆** |  |  |  |  |  |  |  |  |  |  |
|  |  |  |  |  |  |  |  |  |  |  |
| *50S / 100Y* |  | 0.092 · |  |  |  |  |  |  |  |  |
| *50S / 200Y* |  | 0.143 | 0.000 *** |  |  |  |  |  |  |  |
| *100S / 50 Y* |  | 0.299 | 0.508 | 0.001 *** |  |  |  |  |  |  |
| *100S / 100Y* |  | 0.186 | 0.575 | 0.005 ** | 0.986 |  |  |  |  |  |
| *100S / 200Y* |  | 0.379 | 0.006 ** | 0.593 | 0.028 * | 0.024 * |  |  |  |  |
| *200S / 50Y* |  | 0.000 *** | 0.000 *** | 0.000 *** | 0.000 *** | 0.000 *** | 0.000 *** |  |  |  |
| *200S / 100Y* |  | 0.135 | 0.000 *** | 0.779 | 0.000 *** | 0.006 ** | 0.698 | 0.000 *** |  |  |
| *200S / 200Y* |  | 0.000 *** | 0.000 *** | 0.000 *** | 0.000 *** | 0.000 *** | 0.000 *** | 0.000 *** | 0.001 ** |  |
|  |  |  |  |  |  |  |  |  |  |  |

Significance levels: ‘·’ p<0.1 ; ‘*’ p<0.05; ‘**’p<0.01; ‘***’p<0.001

**Table S2.3** Anova model parameter estimates for effects on reproduction of the *dilp2-3,5* knockout, increasing concentration of dietary sugar (S) and yeast (Y) and their interactions. F-values, sum of squares, mean square and p-values are given.

| *Factor* | *F-value* | *Sum of sq* | *Mean sq* | *Pr > \|F\|* |  |
| --- | --- | --- | --- | --- | --- |
|  |  |  |  |  |  |
| *Genotype* | 1564.014 | 92.88 | 92.88 | < 2e-16 | ******* |
|  |  |  |  |  |  |
| *Sugar* | 406.860 | 48.32 | 24.16 | < 2e-16 | *** |
| *Yeast* | 599.170 | 71.16 | 35.58 | < 2e-16 | *** |
|  |  |  |  |  |  |
| *Sugar*Yeast* | 13.935 | 3.31 | 0.83 | 8.60e-10 | *** |
| *Sugar*Genotype* | 26.587 | 3.16 | 1.58 | 1.03e-10 | *** |
| *Yeast*Genotype* | 3.644 | 0.43 | 0.22 | 0.028 | * |
|  |  |  |  |  |  |
| *Sugar*Yeast*Genotype* | 2.440 | 0.58 | 0.14 | 0.049 | * |
|  |  |  |  |  |  |

**S**ignificance levels: ‘·’ p<0.1 ; ‘*’ p<0.05; ‘**’p<0.01; ‘***’p<0.001
