# Supplementary material for: Both overlapping and independent mechanisms determine how diet and insulin-ligand knockouts extend lifespan of Drosophila melanogaster
Source: NPJ Aging Mech Dis. 2017 Feb 20;3:4. doi: 10.1038/s41514-017-0004-0 (PMC5445580; doi:10.1038/s41514-017-0004-0)
Supplement: Supplementary file 6 — Supplementary Data [file 41514_2017_4_MOESM6_ESM.docx]

**Data S1 Materials and Methods**

**Fly stocks and husbandry**

*Dilp2-3,5* mutants were backcrossed for at least 6 generations into the control fly background, outbred *Dahomey* flies carrying the *w1118* mutation (Gronke *et al*., 2010) and kept in population bottles, being refreshed with an additional 2 generations of backcrossing every 6 months. This experiment was performed in 2012. The control flies were housed in population cages with overlapping generations and maintained at standard density in bottles for two generations before the start of the experiment. The experiment was performed at 25°C, 12-hour light⁄dark cycle at 65% humidity on once-mated female flies. Flies were reared at standard density according to the method of Clancy *et al*. (2001), and developed for 10 days on standard food (1SY-A). After emergence, adult flies were transferred to fresh 1SY-A bottles to mate for 48 hours. Under CO_2_ anesthesia female flies were collected and allocated at a density of 10 flies into vials containing one of the nine food treatments.

**Food treatments**

For all food types per liter the following ingredients were added: 15 g agar (Sigma, Dorset, UK), 3 ml propionic acid (Sigma, Dorset, UK), 30 ml Nipagin (Clariant UK Ltd, Pontypridd, UK) in 95% ethanol. The quantities of autolysed Brewer’s Yeast (MP Biomedicals, Solon, OH) and sucrose (Tate & Lyle sugars, London, UK) varied between the food types. Standard food (1SY-A) consisted of 50 gr.l^-1^ sucrose and 100 gr.l^-1^ autolysed yeast. The nine food varied in the combination of sucrose and yeast concentrations as in Skorupa *et al.* (2009), but at concentrations of 50 gr.l^-1^ , 100 gr.l^-1,^ and 200 gr.l^-1^; these diets were chosen because they encompass the S/Y concentrations that maximized lifespan in Bass *et al* (2007) and Skorupa *et al.* (2009), studies which we aimed to use as benchmarks.

**Reproduction assay**

Reproduction was determined by counting the number of eggs laid in 24 hrs per vial. Eggs were counted every week for the first six weeks of adult life. Per week the average egg count per female per vial was calculated and used in the analyses

**Lifespan assay**

For lifespan analysis three times per week flies were placed onto fresh media and for each transfer deaths were recorded.

**Statistical analyses**

To test lifespan effects of, and interactions, between genotype, sucrose and yeast concentration, a cox proportional hazard test was performed. For reproduction, the total egg count data for each vial was summed and log transformed. An analysis of variance (Anova) was performed to test for effects of and interactions between genotype, sucrose and yeast concentration, a t-test was performed to test for differences between single diet treatments within genotype.

**References Materials and Methods**

Bass TM, Grandison RC, Wong R, Martinez P, Partridge L, Piper MDW. Optimization of Dietary Restriction Protocols in Drosophila. *J Geront.* 2007; 10; 1071–1081

Clancy DJ, Kennington WJ. A simple method to achieve consistent larval density in bottle cultures. *Dros. Inf. Serv*. 2001; 84: 168-169.

Gronke S, Clarke DF, Broughton S, Andrews TD, Partridge L Molecular evolution and functional characterisation of Drosophila insulin-like peptides. PLoS Genet. 2010; 6: e1000857

Skorupa DA, Dervisefendic A, Zwiener J, Pletcher SD. Dietary composition specifies consumption, obesity, and lifespan in Drosophila melanogaster. *Aging Cell* 2008; 7: 478–490.
